# Preliminary clinical experience with hyaluronan anti-adhesion gel in arthroscopic arthrolysis for posttraumatic elbow stiffness

**DOI:** 10.1007/s10195-013-0229-z

**Published:** 2013-03-01

**Authors:** Luigi Adriano Pederzini, Luigi Milandri, Massimo Tosi, Mauro Prandini, Fabio Nicoletta

**Affiliations:** Arthroscopic and Sport Medical Center, New Sassuolo Hospital, Via Francesco Ruini 2, 41049 Sassuolo, MO Italy

**Keywords:** Hyaloglide, Hyaluronan, Elbow surgery, Arthrolysis

## Abstract

**Background:**

Loss of motion of the elbow joint is a common finding after elbow trauma. It has been shown that arthroscopic treatment leads to excellent restoration of elbow motion, although it is still a demanding procedure. The aim of our cohort study was to assess clinical outcomes following treatment of posttraumatic elbow stiffness using arthroscopic arthrolysis with or without the associated use of a hyaluronan anti-adhesion gel.

**Materials and methods:**

A cohort of 36 consecutive patients undergoing elbow arthroscopic arthrolysis were enrolled: 17 patients in the hyaluronan gel group and 19 in the control group. The patients underwent prospective control visits 30 and 75 days after surgery. Functional outcome was measured by the range of motion and the Liverpool elbow score (LES), whereas pain and quality of life were evaluated using the visual analogue scale and the SF-36 questionnaire, respectively.

**Results:**

The range of motion and the overall LES score increased over time in both groups. The mean increase over time was statistically significant (*p* < 0.001) in both groups and there was no difference between the groups. There was also no interaction between time and treatment. The percentage of patients who reported pain decreased significantly over time (*p* = 0.0419) in the hyaluronan-treated group (suggesting limited contractions and better comfort during rehabilitation), but not in the control group. The intensity of pain decreased significantly over time in both groups (*p* < 0.0001) without any significant difference between the groups. All the changes in patient quality of life as measured by the SF-36 questionnaire were similar for the two groups of patients. No adverse event or complication related to the application of hyaluronan gel occurred.

**Conclusions:**

Our preliminary clinical experience showed promising results upon the use of hyaluronan gel, considering that it significantly reduced pain in the short term, facilitating a more comfortable rehabilitation. These findings should be confirmed by larger studies.

## Introduction

Stiff elbow can result from several different etiologies, including burns, trauma, spasticity, osteoarthritis, and septic arthritis. The most common of these is trauma to the elbow, in which intrinsic changes set causal conditions in motion. The mechanism of posttraumatic elbow contracture is intra-articular effusion, which induces the elbow joint to develop intra-articular adhesion and capsular thickening, limiting flexion, extension, and pronosupination, thus resulting in posttraumatic elbow stiffness, loss of motion, and considerable impairment in daily life. Established contractures should initially be treated with physical therapy and static progressive splinting. Patients who have failed a minimum of six months of nonsurgical management are candidates for a surgical approach [1–3]. Less invasive techniques for elbow contracture release have been developed in an effort to avoid excessive scarring and soft-tissue trauma, which may contribute to contracture recurrence. More recently, arthroscopic release of posttraumatic elbow stiffness has gained acceptance, and has been shown to lead to excellent restoration of elbow motion [4–7]. Arthroscopic surgery is a demanding procedure that requires great surgical expertise, and a high risk of severe neurovascular complications has been reported in the literature [4].

Recently, an autocrosslinked hyaluronan polymer that acts as an absorbable physical barrier to prevent or reduce postsurgical adhesions in tendon as well as nerve and articular surgery has become available. The gel consists of an autocrosslinked polysaccharide (ACP) that forms when hyaluronan crosslinks without any foreign substances present, so catabolism leads only to hyaluronan. The gel retains all of the properties of HA but enhances its viscoelastic properties and acts as a tissue lubricator, which aids gliding [8]. Preclinical and clinical studies have demonstrated that this mechanical barrier remains in situ during the critical period of adhesion formation and effectively reduces adhesion formation in tendon, nerve, and joint surgery [9–18]. We carried out a cohort clinical trial to assess clinical outcomes after elbow arthroscopic arthrolysis with or without the use of the autocrosslinked hyaluronan gel.

## Materials and methods

A prospective cohort study was conducted from March 2006 to June 2008. Thirty-six consecutive patients who were admitted to our institution were enrolled in the study. Patients undergoing an elbow arthroscopic arthrolysis for posttraumatic elbow stiffness were included and alternatively allocated by the surgeon to the treatment group, where they received intrasurgical application of the autocrosslinked hyaluronan gel (Hyaloglide^®^ from Anika Therapeutics s.r.l., Abano Terme, Italy), or to the control group, where they did not receive any anti-adhesive postsurgical treatment. Inclusion criteria were posttraumatic and postsurgical elbow stiffness, isolated or combined stiffness with an extension gap of less than 20°, flexion contracture of less than 120°, or pronosupination deficit. The exclusion criteria were an arthritic stiff elbow, articular incongruence, steroid therapy in the previous two months, burns, connectivopathy or immunologic disease, diabetes, or alterations in blood coagulation. The study was conducted in accordance with the ethical principles established in the 1964 Declaration of Helsinki and its revisions, and it was approved by the local ethical committee. Written informed consent was obtained from all patients. Safety was assessed by noting any adverse events that occurred during the study. Efficacy outcomes were collected 30 and 75 days after surgery, and were gauged by measuring the range of motion (ROM) using a goniometer and the Liverpool elbow score (LES), a validated elbow-specific instrument combining a patient questionnaire and a clinical evaluation of strength, motion, and ulnar nerve involvement, where 0 indicates the worst function and 10 indicates normal function [19]. A 10 cm visual analogue scale (VAS) was utilized for pain evaluation. In addition, patients were asked to fill in the SF-36 questionnaire before the surgical operation and at the final follow-up visit. The 36 enrolled patients (19 in the control group and 17 in the hyaluronan group; mean age 33.3 ± 10.6 years old) had well-balanced clinical and demographic characteristics at baseline, as summarized in Table 1, except that calcifications occurred more often in the treated group than in the control group. The most commonly involved elbow was the right (52.8 %), and 75 % of the cases did not have ulnar nerve involvement.

**Table 1:** Baseline clinical and demographic characteristics

	Treatment group		Total	*p* value
	Control	Hyaloglide^®^		
Age, mean years (SD)	33.2 (11.4)	33.5 (9.9)	33.3 (10.6)	0.9
Gender, % (SD)				0.24
Female	8 (42.11)	4 (23.53)	12	
Male	11 (57.89)	13 (76.47)	24	
Involved elbow, % (SD)				0.52
Right	11 (57.89)	8 (47.06)	19	
Left	8 (42.11)	9 (52.94)	7	
Ulnar nerve involvement, % (SD)				1
No	14 (73.7)	13 (76.5)	27	
Yes	5 (26.3)	4 (23.5)	9	
Presence of calcification, *n* (%)				0.04
No	12 (63.16 %)	5 (29.41 %)	17	
Yes	7 (36.84 %)	12 (70.59 %)	19	
LES score, mean (SD)	6.54 (1.29)	6.46 (1.35)	6.50 (1.30)	0.85
ROM mean (SD)	86.16 (26.19)	75.88 (24.48)	81.3 (25.6)	0.23
Presence of pain, % (SD)				0.66
No	4 (22.2)	2 (11.8)	6 (17.1)	
Yes	14 (77.8)	15 (88.2)	29 (82.9)	
Intensity of pain, mean (SD)	44.2 (26.9)	35.9 (22.2)	40.0 (24.6)	0.34

### Surgical technique

All surgical procedures were performed by the senior author (LAP). Surgery was performed with the patient under brachial plexus anesthesia in the prone decubitus position with a perineural catheter for postoperative pain control and to facilitate physiotherapy. We performed a neurological check a few hours post-op, between the end of brachial plexus anesthesia and the start of anesthetic drug inflow via catheter. A padded tourniquet was used and the arm was supported in an arm holder. In cases with flexion contracture of the elbow of <110°, an open ulnar nerve neurolysis was performed in order to avoid postsurgical nerve apraxia. We first addressed the posterior aspect of the elbow through posterior portals. Adhesions in the subtricipital space and in the olecranon fossa were broken down with an oscillating shaver, and osteophytes (of the humerus and olecranon) were removed with a high-speed burr. After the posterior joint had been treated, the anterior joint was addressed through anteromedial and anterolateral portals. Through these, a shaver was used to develop the space between the anterior humerus and the anterior elbow capsule. Debridement of medial and lateral gutters, contracture, and chondral problems with the radial head were treated. Osseous components, osteophytes, and ectopic bone (of the coronoid and humerus) were removed using an oscillating shaver or a high-speed burr, maintaining capsule integrity. Finally, an anterior capsulotomy was performed with a duckbill punch. Note that it is very important to completely remove the dissected anterior capsule (capsulectomy) with an oscillating soft-tissue shaver in order to avoid contracture recurrence.

Range of motion of the elbow was assessed, and gentle manipulation was performed if necessary to release any remaining capsular contracture. The tourniquet was deflated and hemostasis performed. Two drains were located in the anterior and posterior compartments of the elbow. Before cutaneous suture, the patients who had been randomly assigned to the treatment group received hyaluronan gel (via a prefilled 2 ml transparent and sterile syringe) under arthroscopic guidance. The gel was applied intraoperatively through a skin portal, with half of the contents of the syringe administered to the posterior compartment and the other half to the anterior one. Due to its high viscoelasticity, the gel did not flow through the drains and did not interfere with the blood suction of the drains. Patients assigned to the control group received no anti-adhesion agent. Lastly, the elbow was placed in a static splint at full extension.

On the first day after surgery, all patients initiated active and active assistive range-of-motion exercises with a physician (supervised and continuous passive motion four times per day for 40 min). Drains were removed 2 days after surgery. Patients undergoing postoperative prophylaxis for heterotopic ossification were administered indometacin (25 mg, three times daily for 2 weeks) in association with gastric protection. At home, all patients were prescribed a daily rehabilitative program involving sessions with the physiotherapist (daily, for at least 60–90 min, for a minimum of 45 days) and sessions on a Kinetek machine (30 min, four times daily for 20 days) for the time needed to obtain the greatest gain of elbow motion.

### Statistical analysis

Data are reported as the mean ± standard deviation or counts and percentages, where appropriate. Continuous variables were compared via the unpaired *t* test, and percentages were analyzed using Fisher’s exact test. Presence of pain at successive visits in the two groups was assessed by performing a chi-square test for a linear trend. A repeated-measures analysis of variance (ANOVA) was performed for the overall LES and for each of the LES sections to verify whether the changes in score differed between the two treatment groups and to examine the changes over time in each treatment arm. Paired *t* tests were used to check for differences between the groups in the changes in eight parameters of the SF-36 questionnaire following surgery. A two-sided *p* value of <0.05 was considered statistically significant. Statistical analysis was performed using SAS^®^ software version 9.1 (SAS Institute Inc., Cary, NC, USA).

## Results

The functional results are reported in Table 2. The ROM (mean arc of flexion–extension) increased over time in both groups. The mean gain in motion was statistically significant over time in both groups (ANOVA, *p* < 0.001) and no difference was observed between the groups (*p* = 0.1452). No interaction was observed between time and treatment (=0.979).

**Table 2:** Range of motion analysis (mean total arc of flexion–extension)

Visit	Control group				Hyaloglide^®^ group			
	*N*	Mean (SD)	Min	Max	*N*	Mean (SD)	Min	Max
Before surgery	19	86.16 (26.19)	50	130	17	75.89 (24.48)	20	110
Visit 1 (30 days postsurgery)	17	108.35 (20.55)	70	137	14	93.93 (27.54)	35	130
Visit 2 (75 days postsurgery)	19	120.53 (17.39)	80	140	17	111.29 (22.89)	65	145

Test of fixed effects	Num *df*	Den *df*	*F* value	*p* value
Repeated measures analysis of variance (dependent variable = mean total arc of flexion–extension)				
Treatment	1	34	2.22	0.1452
Time	2	63	54.01	<0.0001
Treatment × time	2	63	0.02	0.9790

The overall LES score, as shown in Table 3, improved over time in both groups, and the mean increase was statistically significant in both groups (ANOVA, *p* < 0.0001), with similar mean increases seen for both treatment groups (*p* = 0.4351). There was no interaction between time and treatment (*p* = 0.6135).

The number of patients who reported pain at baseline and at follow-up visits is reported in Table 4. The percentage of patients reporting pain decreased over time for both groups, although the decrease was only statistically significant (*p* = 0.0419) in the hyaluronan gel treated group. The intensity of pain decreased significantly over time in both groups (ANOVA, *p* < 0.0001), with no significant difference between the groups (*p* = 0.75). As shown in Table 5, all of the changes in quality of life (as measured by the SF-36 questionnaire) between the last visit and before surgery were similar for the two groups of patients (Table 5).

**Table 3:** Overall LES score

Visit	*N*	Mean	Median	SD	Min	Max
Total						
Before surgery	36	6.50	6.83	1.30	3.22	9.00
Visit 1	32	6.61	6.52	1.25	3.78	8.67
Visit 2	36	8.02	8.19	1.31	4.50	10.00
Control group						
Before surgery	19	6.54	6.83	1.29	3.72	9.00
Visit 1	17	6.85	6.78	1.29	4.50	8.67
Visit 2	19	8.19	8.44	1.36	4.72	9.67
Hyaloglide^®^ group						
Before surgery	17	6.46	6.83	1.35	3.22	7.94
Visit 1	15	6.34	6.33	1.21	3.78	8.44
Visit 2	17	7.84	8.17	1.25	4.50	10.00

Tests of fixed effects	Num *df*	Den *df*	*F* value	*p* value
Repeated measures analysis of variance (dependent variable = overall LES score)				
Treatment	1	34	0.62	0.4351
Time	2	64	37.81	<0.0001
Treatment × time	2	64	0.49	0.6135

**Table 4:** Pain analysis

Patients	Baseline *n* (%)	30 days *n* (%)	75 days *n* (%)
Control group^a^			
No pain	4 (22.22)	2 (11.76)	6 (31.58)
Pain reported	14 (77.78)	15 (88.24)	13 (68.42)
Total	18	17	19

Patients	Baseline *n* (%)	Visit 1 *n* (%)	Visit 2 *n* (%)
Hyaloglide^®^ group^b^			
No pain	2 (11.76)	2 (13.33)	7 (41.18)
Pain reported	15 (88.24)	13 (86.67)	10 (58.82)
Total	17	15	17

^a^Chi-square test for a linear trend: 0.4853, *p* value = 0.4860

^b^Chi-square test for a linear trend: 4.1373, *p* value = 0.0419

**Table 5:** Analysis of the results for the SF-36 questionnaire (eight domains)

	Mean difference (visit 2 − baseline) (95 % CL)		*p* value
	Control (*n* = 16)	Hyaloglide^®^ (*n* = 13)	
Physical functioning	8.46 (1.83–15.09)	3.85 (−4.07–11.76)	0.34
Role-physical limitation	4.69 (−17.18–26.56)	−1.92 (−25.37–21.53)	0.66
Bodily pain	9.75 (−4.54–24.04)	9.92 (−1.73–21.58)	0.98
Perception of general health	−3.56 (−8.14–1.01)	−1.69 (−9.69–6.31)	0.65
Vitality	−0.94 (−7.26–5.38)	5.38 (−3.46–14.23)	0.21
Social functioning	4.69 (−3.33–12.71)	0.96 (−12.29–14.21)	0.59
Role-emotional limitation	−4.17 (−22.37–14.03)	17.95 (−8.84–44.74)	0.14
Mental health	3.73 (−4.05–11.52)	2.77 (−6.51–12.05)	0.86

Four patients (three in the control group and one in the hyaluronan gel treated group) had portal synovial fluid drainage—a frequent surgical event after arthroscopy in the elbow—that completely resolved itself within 20 days after the administration of antibiotics. No other complication or adverse event related to the hyaluronan gel occurred during the study.

## Discussion

Loss of motion is a common complication after elbow trauma and can significantly interfere with the ability of the patient to perform activities of daily life [2]. Nonsurgical treatment, including physiotherapy and static splinting, can restore a functional arc of motion in some patients, but arthroscopic capsular release of the elbow has been shown to be a safe and reliable treatment for patients with a posttraumatic elbow contracture. It is a technically demanding operation, but it can improve the elbow’s arc of motion, as recently demonstrated [4]. Our scientific interest was drawn to a recent developed autocrosslinked hyaluronan absorbable physical barrier, hyaluronan gel, obtained via a chemical reaction that results in the formation of intra- and/or intermolecular bonds between the hyaluronic acid molecules [16]. As no bridging molecules are involved in the reaction, the main characteristic of this gel is that it retains all of the properties of native HA but shows increased viscoelasticity, enabling the gel to remain in situ for the time taken for adhesions to form before being completely reabsorbed [8, 20–24]. Hyaluronic acid (HA) is a ubiquitous molecule found in all living species, from bacteria to humans. it is present in high concentrations (0.3–0.5 %) in the synovial liquid of the joints and in tendon sheaths. HA also plays a fundamental role as a modulator in several reconstructive biological processes, such as wound healing (during both the inflammatory and exudative phases) [24]. Since the gel has been shown to prevent or reduce postsurgical adhesions in tendon, nerve, and articular surgery [9–18], we decided to investigate its utility in the elbow joint.

In our small cohort study, the product had a safety profile indicating the absence of any adverse events or complications related to its use as anti-adhesive agent. In terms of functional outcomes, both the ROM recovery and the LES scoring system improved during the study in both groups from presurgery to last follow-up visit, consistent with the clinical relevance and validity of our arthroscopic surgical procedure and the results of recent findings [4, 25, 26]. The quality of life SF-36 questionnaire—a tool for measuring health status that provides information about health-related quality of life via eight parameters: physical function, role-physical limitation linked to physical problems, bodily pain, perception of general health, vitality, social functioning, role-emotional limitation linked to emotional problems, and mental health—was assessed for all patients, and all changes between the last visit and the baseline values were found to be similar for the two treatment groups.

The most important outcome of our clinical study was that the percentage of patients reporting pain decreased significantly in the hyaluronan gel group. This finding suggests that the intra-articular presence of hyaluronan gel may have caused a reduction in the recurrence of residual adhesions, thus improving tissue sliding. This may eventually lead to a reduced pain sensation but not increased articulation. Experiencing a significantly reduced pain sensation where the soft-tissue refection occurred is an important factor. Indeed, a reduced pain sensation in the phases immediately after the surgery diminishes antalgic-related contractions, favoring rehabilitative treatment. This ultimately results in increased comfort during physiotherapy.

The authors acknowledge that the small sample size is a weakness of this cohort study, but the results are promising for the use of hyaluronan gel in the prevention of postsurgical adhesions in elbow surgery, and these results should be confirmed in larger studies.
